# Convergence of Cells from the Progenitor Fraction of Adult Olfactory Bulb Tissue to Remyelinating Glia in Demyelinating Spinal Cord Lesions

**DOI:** 10.1371/journal.pone.0007260

**Published:** 2009-09-29

**Authors:** Eleni A. Markakis, Masanori Sasaki, Karen L. Lankford, Jeffery D. Kocsis

**Affiliations:** 1 Department of Comparative Medicine, Yale University School of Medicine, New Haven, Connecticut, United States of America; 2 Department of Neurology and Center for Neuroscience and Regeneration Research, Yale University School of Medicine, New Haven, Connecticut, United States of America; 3 Rehabilitation Research Center, VA Connecticut Healthcare System, West Haven, Connecticut, United States of America; Tokyo Medical and Dental University, Japan

## Abstract

**Background:**

Progenitor cells isolated from adult brain tissue are important tools for experimental studies of remyelination. Cells harvested from neurogenic regions in the adult brain such as the subependymal zone have demonstrated remyelination potential. Multipotent cells from the progenitor fraction have been isolated from the adult olfactory bulb (OB) but their potential to remyelinate has not been studied.

**Methodology/Principal Findings:**

We used the buoyant density gradient centrifugation method to isolate the progenitor fraction and harvest self-renewing multipotent neural cells grown in monolayers from the adult green-fluorescent protein (GFP) transgenic rat OB. OB tissue was mechanically and chemically dissociated and the resultant cell suspension fractionated on a Percoll gradient. The progenitor fraction was isolated and these cells were plated in growth media with serum for 24 hrs. Cells were then propagated in N2 supplemented serum-free media containing b-FGF. Cells at passage 4 (P4) were introduced into a demyelinated spinal cord lesion. The GFP^+^ cells survived and integrated into the lesion, and extensive remyelination was observed in plastic sections. Immunohistochemistry revealed GFP^+^ cells in the spinal cord to be glial fibrillary acidic protein (GFAP), neuronal nuclei (NeuN), and neurofilament negative. The GFP^+^ cells were found among primarily P0^+^ myelin profiles, although some myelin basic protein (MBP) profiles were present. Immuno-electron microscopy for GFP revealed GFP^+^ cell bodies adjacent to and surrounding peripheral-type myelin rings.

**Conclusions/Significance:**

We report that neural cells from the progenitor fraction of the adult rat OB grown in monolayers can be expanded for several passages in culture and that upon transplantation into a demyelinated spinal cord lesion provide extensive remyelination without ectopic neuronal differentiation.

## Introduction

We have shown in our previous work [1], [2] that neural progenitor cultures from a number of different adult brain regions converge to a similar morphology and potential *in vitro*. Cells from regions as distinct in function and embryonic origin as the hypothalamus and the hippocampus yield progenitor cultures that are proliferative in the presence of basic fibroblast growth factor (b-FGF), self-renewing, and capable of generating cells of all three neural lineages. In culture, differentiated progenitors isolated from the adult hippocampus (an alar plate derivative) are able to produce the classic hypothalamic (basal plate derivative) neuroendocrine peptides oxytocin, vasopressin, thyrotropin-releasing hormone, growth hormone-releasing hormone, somatostatin, corticotropin-releasing hormone, and gonadotropin-releasing hormone (GnRH; [2]). This is particularly noteworthy in the case of GnRH because cells that produce this neuropeptide *in vivo* are not generated by the proliferative neuroepithelium of the forebrain - they are generated in the olfactory placode.

Progenitor cells can be valuable substrates for novel glial cell generation. While oligodendrocytes are not the predominant cell type found in differentiated progenitor cultures, new oligodendrocytes are generated by these cells, and some progenitors have been able to remyelinate demyelinated axons [3]–[6]. The neurogenic hippocampus and subependymal zone of the lateral ventricles [7], [8], [Bibr pone.0007260-Craig1] [9: for a review see 10] were the initial targets for progenitor isolation, but subsequent work has uncovered progenitors in other brain regions including the isocortex, optic nerve, hypothalamus; each of these areas has yielded self-renewing cultures capable in the presence of b-FGF of generating cells of all three neural lineages [1], [2].

The adult olfactory bulb (OB) is a novel site for progenitor isolation, but portions of it have been found to contain neural progenitor cells [11]. The OB is the destination of neural progenitor cells generated in the subependymal zone [12], [13] that migrate down the rostral migratory stream and generate new interneurons in the internal granule layer of the OB. While multipotent neural progenitors have been isolated from the OB [14] the potential of transplanted neural progenitor cells derived from the OB to remyelinate demyelinated axons has not been addressed.

Our work indicates that multipotent cells isolated from the progenitor fraction of different brain regions have similar potential *in vitro*. In order to detect convergence of their potential *in vivo*, we grafted such cells isolated from the adult rat OB into a well characterized aglial lesion paradigm in the rat spinal cord, and compared our results to those obtained from multipotent progenitors isolated from other central nervous system (CNS) regions (spinal cord for example, [15]). We isolated cells from the progenitor fraction (using previously established terminology [16]) from transgenic GFP-expressing adult rat OB (OB cells) using a buoyant density gradient centrifugation method [1], propagated cells in monolayers, and characterized them immunocytochemically. We transplanted these cells at P4 into demyelinated rat spinal cord lesion. Grafted GFP^+^ cells produced robust, extensive remyelination of the spinal cord with a largely peripheral pattern of myelin. These results indicate that cells from the OB progenitor fraction are capable of remyelinating axons in the CNS with a predominantly peripheral Schwann-like pattern of myelination, similar to that reported for other central neural progenitors, and indicate that the OB may provide a rich source of cells for remyelination.

## Materials and Methods

### Ethics Statement

Experiments were performed in accordance with National Institutes of Health guidelines for the care and use of laboratory animals, and the Veterans Affairs Connecticut Healthcare System Institutional Animal Care and Use Committee approved all animal protocols.

### Isolation of Cells from the Progenitor Fraction

The methods used to isolate cells from the progenitor fraction of the OB are similar to those used in hippocampal progenitor isolation, and have been described in detail elsewhere [17], [18], [1], [2]. In brief, seven week old GFP^+^ transgenic rats (CZ-004, SD-Tg(Act-EGFP)CZ-004Osb; SLC, Shizuoka, Japan; see [19]) were anesthetized and placed in the frame of a stereotaxic instrument (David Kopf, Tujunga, CA). Skin was incised and the cranium exposed, and a window of bone removed over the rostral cerebral cortex and OB. The bulbs were separated from the cortex using Vannas scissors, and bulb tissue was removed with forceps, and placed in a tube filled with sterile, chilled DMEM/F12 media. Bulb tissue from 10 rats, either transgenic GFP^+^ rats (for transplantation) or Sprague Dawley rats (culture immunocytochemistry) was pooled per preparation. A total of eight OB cultures were generated and studied. Tissues were minced and digested in a solution of papain (2.5 U/ml; Worthington, Freehold, NJ), neutral protease (1 U/ml Dispase; Boehringer Mannheim, Indianapolis, IN), and DNase (250 U/ml, Worthington) in HBSS. Cells were spun and washed twice in DMEM/F12 (1∶1) with 10% FBS. The resulting cell suspension was layered onto a 30/40/50 Percoll gradient made of Percoll and saline (40%) or media (30% and 50%). The cell suspension was fractionated by centrifugation for 30 min at 20,000×g at 18°C. The progenitor fraction (40%/50% interface) was harvested and washed free of Percoll and plated on polyornithine–laminin-coated dishes in growth medium consisting of DMEM/F12 (1∶1) high glucose with N2 supplement (Invitrogen, Gaithersburg, MD) and 20 ng/ml recombinant human fibroblast growth factor-2 (b-FGF) prepared in E. coli (Peprotech, Rocky Hill, NJ). Cells were maintained for their first 24 hr in this growth medium, plus 10% FBS, and subsequently grown in serum-free media. Cells at passage 4 (P4) were chosen for analysis and grafting. Remaining cultures were differentiated in serum media containing all-trans-retinoic acid (100 ng/ml) and forskolin (5 µM).

### Immunocytochemistry

Cells were plated onto polyornithine/laminin coated 4-well glass chamber slides where they were fed and established for 48 hrs. Media was suctioned off and cells were rinsed with sterile PBS, then covered with ice-cold 4% paraformaldehyde in 0.14 M Sorensen's phosphate buffer, pH 7.4 for 10 minutes. Paraformaldehyde was suctioned off and slides were stored in PBS at 4°C until they were stained. Immunostaining began with removal of the buffer and addition of KPBS buffer containing a permeabilization agent, Triton X100 (0.3%), and a blocking agent, normal donkey serum (1%) to the wells. Triton buffer was suctioned off the wells after one hour and primary antibodies in optimal dilutions in KPBS were added to each well. Slides in primary antibody were incubated at 4°C for three days, then primary was suctioned off and slides were rinsed with KPBS and incubated with secondary antibodies at room temperature for one hour. Following two final rinses in KPBS, gaskets were removed from the slides and slides were coverslipped using PVA-DABCO mountant (see [2]).

### Demyelinating lesion and grafting

Demyelinating lesions were performed on 16 rats Sprague Dawley rats (8 week-old, 150–175 g), 10 of which also received cell grafts. A focal demyelinating lesion was created in the dorsal column of the rat spinal cord with X-irradiation and ethidium bromide injection (X-EB) as previously described [20], [21], [22]. Briefly, rats were deeply anesthetized with ketamine (75 mg/kg) and xylazine (10 mg/kg), and a 40 Gy surface dose of X-irradiation was delivered through a 2×4 cm opening in a lead shield (4 mm thick) to the spinal cord caudal to T8 using a Siemens (Erlangen, Germany) Stabilipan radiotherapy machine (250 kV, 15 mA, 0.5 mm Cu; 1 mm Al filters; source skin distance, 28 cm; dose rate, 220.9 cGy/min). Three days after irradiation, rats were anesthetized, a laminectomy was performed at T9, and injections of 0.5 µl (0.3 mg/ml EB in saline) were made at depths of 0.7 and 0.5 mm at three sites 2 mm apart using a drawn-glass micropipette. Three days after EB injection, injections of 0.5 µl of OB cells at P4 suspended in sterile HBSS (3.0×10^4^ cells/µl) were given at depths of 0.7 and 0.5 mm into the center of the X-EB-induced lesion. Rats were immunosuppressed using cyclosporine A (10 mg/kg, Sandoz Pharmaceuticals) beginning the day before transplantation, and daily thereafter until perfusion. Grafted spinal cords were processed for plastic section and electron microscopy (n = 4), immunohistochemistry (n = 4), and immunoelectron microscopy (n = 2) 3 weeks later.

### Ultrastructural analysis of myelin

Three weeks after transplantation, rats were deeply anesthetized with sodium pentobarbital (60 mg/kg, i.p.) and perfused with 2% paraformaldehyde plus 2% glutaraldehyde in 0.14 M Sorensen's phosphate buffer, pH 7.3. Spinal cords were excised, stored overnight in fixative, cut into 2 mm segments, notched to indicate orientation, postfixed with 1% osmium (Polysciences, Warrington, PA) for 4 hrs, dehydrated, and embedded in Epox-812 (Ernest F. Fullam, Latham, NY) using standard plastic embedding protocols. Semithin sections (1 µm) were collected along the length of the cord and counterstained with methylene blue and azure II (0.5% each in 0.5% borax). Ultrathin (70 nm) sections of the lesions were counterstained with uranyl acetate and lead citrate and examined with a Philips 300 electron microscope operated at 80 kV. One layer of myelin or lamella was operationally defined as two electron dense bands separated by an electron lucent band. To determine the periodicity of the myelin, the distance between the centers of the innermost and outermost of a series of at least six electron dense bands was measured from scanned EM negatives (magnification, ≥10,000x) using Novaprime Bioquant (Nashville, TN) software, and the periodicity was calculated by dividing this distance by the numbers of electron dense bands minus one. Data were expressed as mean±SEM.

### Immuno-electron microscopy

Animals were deeply anesthetized (50 mg/kg sodium pentobarbital, i.p.) and perfused transcardially with PBS followed by 4% paraformaldehyde/0.02% glutaraldehyde in PBS. Spinal cords were excised, postfixed overnight in 4% paraformaldehyde, and embedded in 3% agar for vibratome sectioning. Free-floating sections (150 µm thick) were incubated in 2% normal goat serum for 30 minutes and then in rabbit anti-GFP antibody (1∶2000; Chemicon) overnight at 4°C. The sections were incubated overnight with a biotinylated anti-rabbit secondary antibody (Sigma, St. Louis, MO) and then incubated for 1 hour using a Vectastain Elite ABC kit (Vector Laboratories, Burlingame, CA) similar to that described by Sasaki et al. [19]. The sections were then postfixed with 1% osmium tetroxide for 4 h, dehydrated in graded ethanol, and embedded in Epox-812 (Ernest Fullam, Latham, NY). Ultrathin sections were cut as described above.

### Immunohistochemistry

Spinal cords of transplanted rats were processed for immunocytochemistry as described previously [23]. Briefly, rats were deeply anesthetized with ketamine/xylazine and perfused transcardially, first with PBS and then with ice-cold 4% paraformaldehyde in 0.14 M Sorensen's phosphate buffer, pH 7.4. Spinal cords were excised and placed in fresh fixative to achieve a total fixation time of 25 min. Spinal cords were rinsed several times with PBS and immersed in 30% sucrose in PBS overnight at 4°C. Fifteen micrometer coronal sections, or longitudinal sections of the dorsal columns were cut on a cryostat and mounted on Fisher Superfrost Plus glass slides. Sections were processed for single immunolabeling for GFAP, MBP, NF and P0. After multiple rinses in PBS, sections were incubated in PBS with 5% normal goat serum, 1% BSA, and 0.1%Triton X-100 for 20 minutes, followed by primary antibody incubation for 24 hrs at 4°C. Sections were rinsed and incubated in secondary antibody for one hour at room temperature. Slides were coverslipped after several rinses in PBS, and examined under a confocal microscope.

### Counts

Cells in the grafted spinal cord were quantified using field counts of immunolabeled tissue. Axon profiles were identified in tissue sections (n = 4) of random non-contiguous fields, in tissue labeled with the antibody to P0 or MBP, and profiles were scored as immunolabeled or immunonegative. In P0-labeled tissue, 333 axon profiles were counted and scored. In MBP-labeled tissue 209 axon profiles were counted and scored.

### Microscopy

Tissue sections and cell slides were examined under a fluorescence scope equipped with confocal lasers (Zeiss LSM510, Thornwood, NY). Images were captured using LSM 510 software (Zeiss, Thornwood, NY) and arranged using Adobe Illustrator and Photoshop (Adobe Systems, San Jose, CA).

### Antibodies

The antibody to P0 was obtained from Dr. Juan Jose Archelos, University of Graz, Graz, Austria. It was used at a concentration of 1∶150. Commercially available antibodies used were: mouse anti-A2B5 (1∶100; Millipore MAB312, Temecula, CA); mouse anti-GFAP (1∶1000; Sigma G3893 Saint Louis, MO); mouse-anti MBP (1∶500; Sternberger SMI94 and SMI99, Lutherville, MD); mouse anti-nestin (1∶500; Millipore MAB353 Temecula, CA); mouse anti-NeuN (1∶100; Millipore MAB377 Temecula, CA); mouse anti-O4 (1∶10; Millipore MAB345 Temecula, CA); mouse anti-RIP (1∶5000; Millipore MAB1580 Temecula, CA); mouse anti-TUJ-1 (1∶500; Covance MMS-435P Dedham, MA); mouse anti-vimentin (1∶20; Abcam AB8978 Cambridge, MA); rabbit anti-P75 (1∶250; Millipore AB1554 Temecula, CA). Secondary antibodies used were: goat anti-rabbit IgG-Cy3 (1∶2000; Amersham Biosciences, Piscataway, NJ); goat anti-mouse IgM Alexa-Fluor 633, goat anti-mouse IgG-Alexa Fluor 633 and -Alexa Fluor 546 (1∶1000; Invitrogen, Eugene, OR); Donkey anti-mouse IgG-Rhodamine RedX (1∶250; Jackson ImmunoResearch #715-295-151 West Grove, PA) and Donkey anti-mouse IgM-Rhodamine RedX (1∶250; Jackson ImmunoResearch #715-296-020 West Grove, PA).

## Results

### Cells from the OB progenitor fraction

**Figure 1:**
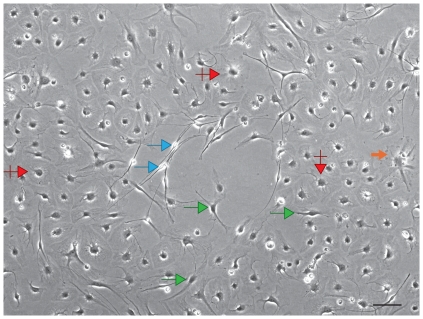
Photomicrographs of cells *in vitro*. A. Phase contrast light photomicrograph of cells isolated from the progenitor fraction of the adult rat OB at P5. Cells are of four basic morphologies: round cells with central nuclei (red crossed arrow), bipolar with long processes (green arrows), large, amorphous cells with eccentric nuclei (thick orange arrow), and ovoid nucleus with long, fine processes (blue arrows). The scale bar is: 10 µm.

OB cells from the progenitor fraction were plated in serum-supplemented media onto polyornithine-laminin coated plates. Within 24 hrs the serum media and all non-adherent cells were suctioned off the plate. Cultures were fed with a 1∶1 mixture of DMEM/F12 supplemented with N2, conditioned media from established progenitor cultures, and b-FGF. The cells that adhered to the plate were of four basic morphologies (Fig. 1): large round cells with central round nuclei, prominent bipolar cells with thick processes and dense nuclei, large amorphous cells with eccentric nuclei with radial processes, and small bipolar cells with translucent nuclei and long fine processes. Cultures expanded rapidly in the presence of b-FGF and reached confluence in less than one week. Confluent cultures were passaged 1∶2 and continued to expand for at least eight passages; cells at P4 were chosen for grafting.

As might be expected of tissue that lies at the end of the rostral migratory stream, the OB is a potent source of proliferative cells. Given the relatively small volume of tissue from which the cultures were generated, the OB cultures were exceptionally robust, outpacing hippocampal progenitor cultures isolated and plated in parallel. Thus while initial plating densities differed, with hippocampal cultures generated from much more tissue (volume of hippocampal tissue averaged 16 ml, while OB tissue averaged 7 ml), plating at roughly twice the density of OB cultures, the OB cultures were considerably more proliferative. To quantify the rate of proliferation confluent dishes at P4 were studied for population doublings. A single confluent dish was passaged into two dishes (1∶2; P5) and after a number of days in culture two dishes were passaged into four (1∶2; P6). Hippocampal cultures spent an average of 4.6 days in culture between population doublings, while OB cultures averaged only 3 days, or 35% less time in the dish between passages.

**Figure 2:**
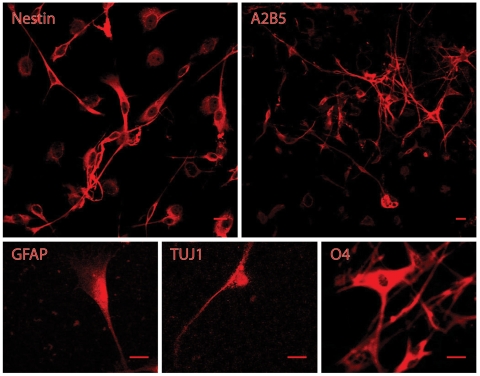
Confocal photomicrographs of immunolabeled cells from OB cultures. OB cells at P4 labeled for a number of antigens, including nestin, A2B5, GFAP, TUJ-1 and O4 indicating that immature proliferative cells and cell of all three neural lineages can be generated from the progenitor fraction in these cultures. The scale bars are 10 µm.

In order to avoid frank transformations and aneuploidy [24], OB cultures were not immortalized nor were they extensively expanded. We studied the bulk population, not clones, because we wished to eliminate the possibility that we might report on a phenomenon that is limited to a rare sub-population of cells. Cultures at P4 were fixed and immunocytochemistry for a number of antigens was performed. Cultures generated cells that labeled with the antibody to vimentin (not shown), nestin, A2B5 (Fig. 2), and differentiated cell types (Fig. 2) including oligodendrocytes (O4, RIP not shown), astrocytes (GFAP) and neurons (type III b-tubulin (TUJ-1), NeuN not shown). OB cultures confirm our previous observation that cells from the progenitor fraction of diverse brain regions tend to converge to “standard” progenitor culture morphology, generating neurons and both types of glial cells [1] and those progenitors are largely vimentin positive (87%), have a substantial O4 positive population (24%) and contain some nestin positive cells (0.1%), while containing few cells of differentiated neural cell types (GFAP, TUJ-1 0.1%, NeuN<0.1%). OB cultures did not label with the antibody to P75, and when differentiated, lost nestin immunoreactivity and contained groups of NeuN^+^ cells (not shown).

Passage 4 (P4) was chosen for analysis and transplantation because the likelihood that any cell examined at P4 was actually a cell isolated in the initial cell preparation is infinitesimally small. Furthermore, mature neurons do not passage well, so even if our initial plating were contaminated with neurons, they would not persist to P4. Thus we conclude that the cells of neuronal phenotypes demonstrated in undifferentiated cultures were generated by the cultures. The cultures were proliferative and self-renewing in the presence of b-FGF, and generated cells of all three neural lineages.

### Cell transplantation

**Figure 3:**
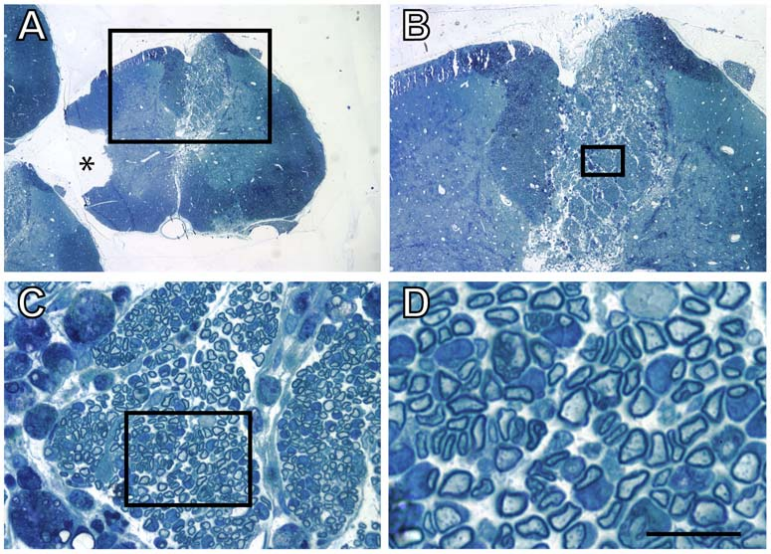
Plastic section light microscopy of grafted X-EB lesioned spinal cord. Progressive magnifications of semithin plastic sections through X-EB lesioned spinal cord 3 weeks after grafting with OB cells show the lesion site at low power in panel A, filled with grafted cells. The fiduciary notch (asterisk) identifies the left side of the spinal cord. Higher magnification of the boxed regions in subsequent panels shows myelinated axons, many of which are associated with large cytoplasmic and nuclear regions characteristic of peripheral myelination. The scale bar is 1 mm in A, 400 µm in B, 40 µm in C, and 10 µm in D.

OB cells generated from GFP^+^ rats were transplanted into X-EB demyelinated lesions of the dorsal funiculus of the spinal cord of wild-type Sprague-Dawley recipients three days following the administration of ethidium bromide. Animals were perfused three weeks after transplantation. We initially evaluated OB-transplants using semithin plastic section light microscopy. Plastic sections make identification of the lesion/transplant site unambiguous (Fig. 3) at low power. Progressive magnifications of the site revealed numerous myelinated axon profiles within the lesion zone (Fig. 3). At highest magnification, cell bodies exhibited the characteristic signet ring profiles of peripheral myelin (Fig. 3).

**Figure 4:**
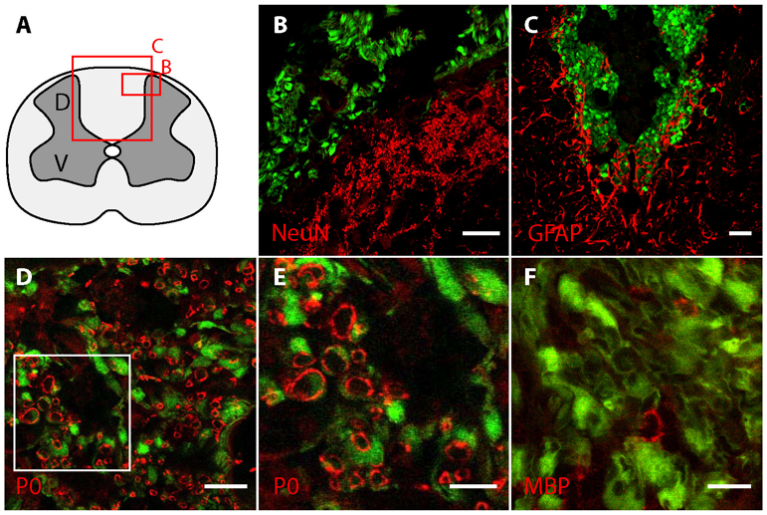
Confocal photomicrographs of immunohistochemistry performed on grafted X-EB spinal cord tissue. The illustration in A shows the location of panels B and C. GFP^+^ cells were grafted into the X-EB lesion and survived well. NeuN^+^ profiles in the dorsal horn were found among GFP^+^ cells. Grafted cells did not differentiate into neurons, as GFP^+^ cells did not label for NeuN (B). GFAP^+^ cells were observed primarily outside the lesion; transplanted cells did not assume a GFAP^+^ profile, as GFP^+^ cells did not also label with the antibody to GFAP (C). Progressive magnifications of the boxed region of GFP^+^ OB cells (D, E) show close apposition of GFP^+^ cells with P0-positive myelin. Some MBP^+^ profiles were also found among grafted cells, indicating the generation of some central myelin in the graft site (F). Scale bar is: 80 µm in B and C, 40 µm in D and F, and 20 µm in E.

Subsequent immunohistochemical staining evaluated with confocal microscopy revealed GFP^+^ cells surviving well within the lesion, that were found among cells that were neurofilament^+^, TUJ-1^+^(not shown), or NeuN^+^ (Fig. 4B) but the GFP^+^ cells did not label for those markers, thus the cells did not differentiate into neurons after transplantation into the lesion. Immunohistochemistry for GFAP also showed no co-localization of GFP and GFAP, indicating that the cells had not differentiated into astrocytes (Fig. 4C). As the transplanted tissue contained novel myelin rings, we immunolabeled for O4, a marker of immature oligodendrocytes, and found no double labeling with GFP (not shown), although this does not preclude the possibility that O4^+^ cells in culture contributed to the remyelination seen. The low-affinity neurotrophin receptor p75, a marker for immature Schwann cells, was not found within the lesion (not shown).

We immunostained grafted tissue for peripheral (P0 [25]) and central (MBP) myelin and found the large majority of myelin-ring profiles appeared to be P0^+^ (Fig. 4D–F). We quantified this observation by performing counts of axonal profiles, recognized as dark circular regions surrounded by GFP^+^ cells, in tissue labeled for P0 or MBP through the grafting site. In P0-labeled tissue, 333 identified axonal profiles were counted, of which 283 (83%) were P0^+^. In MBP-labeled tissue 209 axonal profiles were counted, and 8 (3.8%) were MBP^+^.

### Ultrastructural analysis of remyelinated axons

**Figure 5:**
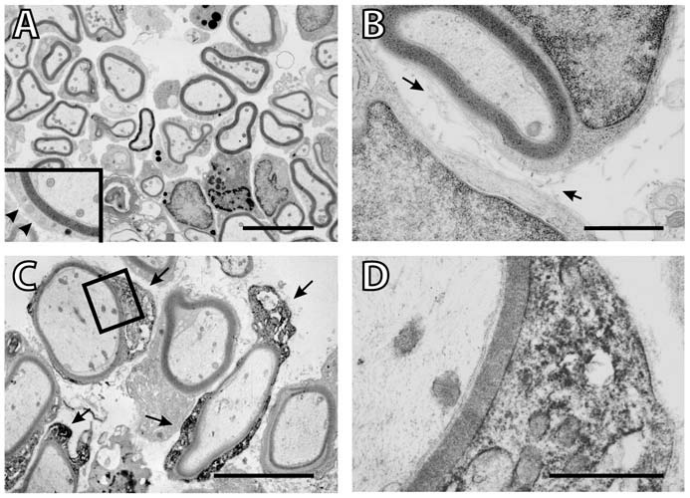
Ultrastructural characteristics of remyelinated axons in the dorsal funiculus of the spinal cord after OB cell grafting. In panels A and B, myelin-forming OB cells had large nuclear and cytoplasmic regions characteristic of peripheral myelin-forming cells; collagen (arrows in B) was detected in the extracellular space. Examination at higher magnification shows basal lamina surrounding the myelin-forming cells (arrowheads in A inset). C. Immunoelectron microscopy for GFP shows GFP^+^ cells remyelinating the demyelinated axons. Counterstaining for these sections was minimal and dark staining shows electron dense immunoperoxidase reaction product. D. Boxed area from C shows the electron dense reaction product in the cytoplasm and nuclei of cells forming myelin. The scale bar is 5 µm in A, 1 µm in B, 5 µm in C, and 1 µm in D.

To determine the structural characteristics of the novel myelin, we examined grafted cords at the ultrastructural level three weeks after OB cell transplantation. Compact myelin was observed at this time point (Fig. 5A), with basal lamina surrounding the myelin-forming cells (inset Fig. 5A); collagen was detected in the extracellular space (Fig. 5B, arrows). The mean number of lamellae of OB cell myelinated axons is 16.9±0.65 (n = 61). The periodicity of OB cell myelin was 12.7±0.08 nm: (n = 62). The myelin generated by these cells has similar characteristics to that generated with olfactory ensheathing cell (OEC) grafts in the same model system at the same time point [22].

### Immuno-electron microscopy

To determine whether the cells generating the P0^+^ myelin rings we observed in transplanted animals were GFP^+^ we performed immunoelectron microscopy using the antibody to GFP. Cell bodies giving rise to the myelin profiles encircling host axons within the lesion were immuno-positive in their cytoplasm and their nuclei (Fig. 5C, D), thus transplanted GFP^+^ cells gave rise to myelinating cells. GFP immunopositive somata were in close apposition to host axons, and intense GFP reaction product was evident in the cytoplasm of cells that formed multi-laminate structures characteristic of myelin (Fig. 5C, D). The myelin generated by GFP^+^ cells was of a peripheral pattern.

## Discussion

We isolated and characterized cells isolated from the progenitor fraction of the adult rat OB. These cells are highly proliferative and self-renewing in the presence of b-FGF, and generate cells that are immunocytochemically positive for nestin, vimentin, A2B5, and markers of all three neural lineages: neurons (TUJ-1), astrocytes (GFAP), and oligodendrocytes (O4). To determine the utility of OB cells in generating novel myelin, we grafted them into an X-EB-induced demyelinating lesion in the spinal cord. This lesion model produces an aglial zone where both astrocytes and oligodendrocytes are killed. The X-irradiation kills NG2+ oligodendrocyte progenitor cells within the radiation field thus eliminating endogenous remyelination for nearly 2 months [26]. Three weeks after OB cells were introduced into the aglial zone identified grafted cells myelinated axons within the lesion. They did not become neurons or astrocytes; grafted OB cells effected extensive remyelination of axons with a predominantly peripheral pattern.

The definition of a neural progenitor cell can be somewhat ambiguous. The terminology within the literature varies over time and between groups; the name refers to a population of cells that are highly proliferative, self-renewing, and multipotent, generating cells of diverse morphologies and antigen expression. No definitive marker has been identified for progenitor cells - although some erroneously believe nestin to be a definitive progenitor marker. Other researchers using cells isolated in the same way as ours refer to them as progenitor cells [2] or precursor cells [16]. We identify our cells as cells isolated from the progenitor fraction (or simply OB cells), because that fraction has been defined in the literature [2], [16] as the 40%/50% interphase, and gives rise to cells that are highly proliferative in the presence of b-FGF and generate cells of all three neural lineages. Because we cannot assess the extent of self-renewal in low passage cultures, we cannot be certain of the degree of “stem-ness” of the cells we isolated.

### OB cells

Cells can be isolated from the progenitor fraction of adult OB, just as they can from other brain regions be they actively neurogenic or non-neurogenic in the adult [1], [27], [11]. The anatomy of this region makes it a particularly good target for proliferative cell isolation for two reasons: 1) its development reveals a ventricular zone that makes it uniquely poised to be a progenitor-rich zone, and 2) it is the destination of neural progenitor cells and newly generated neurons from the subependymal zone.

In development the OB contains a ventricular zone, and ventricular zones are the hot-spots of cell generation *in embryo*, and contain the residue of cell proliferation in adult neurogenesis. The OB is generated from an outpocketing of the floor of the developing telencephalon. The base of the cell pocket elongates to generate the olfactory tract. The developing OB contains a ventricular zone that is ultimately obscured by the fusion of opposing walls, although modified epithelial cells may occasionally be recognized at the zone of fusion in adult. Neurons in the developing OB are generated in a sequential order, projection neurons first (mitral then tufted cells), interneurons last [28]. The projection neurons are generated before the OB is formed, and they probably arise from the proliferative neuroepithelium at the base of the telencephalon. This neuroepithelium transitions into ependymal cells by the time interneurons are generated, and the subependymal zone (SEZ) continues to generate these cells, throughout life.

In adult, the fusion of the olfactory ventricles is recognized as the rostral-most extension of the SEZ (described by Westergaard [29]) that extends caudally to the anterior wall of the lateral ventricle. The SEZ is a feature conserved throughout mammalian evolution, although it is not always easily seen in rodent; rodent neuroscientists have come to refer to the region as the subventricular zone (SVZ), although the SVZ is a classically defined feature of the embryonic neural tube that is not present in adult brains. The rostral migratory stream (RMS) – also conserved through evolution [30] - shuttles proliferative neural progenitor cells rostrally to become new interneurons in the OB; these new neurons do incorporate into circuitry and are functional, as are the newly generated neurons in the dentate gyrus of the hippocampus [31], however large numbers of undifferentiated progenitor cells are continually shuttled down the SEZ to the OB along with new neurons [32].

We suspect that the SEZ is the source of the cells we have isolated. However, it is possible that there are quiescent neural progenitor cells in the OB, left over from development that may comprise part of our culture population. Another possibility is that culture conditions and mitogens can cause cells that have exited cell cycle, to re-enter it. In fact this has been shown to work for committed oligodendrocyte precursors [33], although not in our culture conditions. There is some confusion, arising perhaps from conflicting terminology in the literature, about the origin of new neurons that become incorporated into the circuitry of the adult OB. Some investigators refer to the bulb proper as a neurogenic region (see for example, [14], [34]. A cell originating from within the adult OB that generates new neurons *in vivo* has not been described. A large number of cells generated by the SEZ migrate down the RMS to the OB, but this is not a homogeneous population (see [35]), cells are not terminally differentiated on their arrival to the OB, and remain mixed with progenitors (for a review, see [32]).

### Olfactory Ensheathing Cells

Our OB cells are not olfactory ensheathing cells (OECs). There have been several reports describing the utility of OECs in effecting remyelination of demyelinated spinal cord [36], [37], [38], [19], [22], [39]. OECs transplanted into in the X-EB model remyelinate axons with a peripheral pattern of myelination similar to that described in the present study.

The OB is an isocortex; the outermost layer (layer 1), the olfactory nerve fiber layer, is the zone through which the olfactory sensory neurons located in the nasal mucosa, send bundles of olfactory nerve fibers into the OB. OECs are a specialized type of glia residing in layer 1 [40] that cloak novel olfactory receptor axons as they grow into the nerve fiber layer. OEC cultures are generated from neonatal rats and are established by microdissection of the olfactory nerve layer from the OB and repeated trituration of the tissue into single cell suspensions [41], [37]. Our tissue harvest starts with adult tissue and uses the entire OB, so there is the potential for OEC contamination of the progenitor cell fraction.

We have several lines of evidence to indicate that the remyelination we observed did not arise from OECs, but from a distinct cell population. OECs have a well-characterized immunocytochemical profile [42], [43], [44] and they express the low affinity neurotrophin receptor p75 *in vitro*. Our cultures did not label with the antibody to p75. The buoyant density centrifugation method used to generate our cultures relies on the fact that differentiated cells have more cytoplasm, hence a different buoyant density, than progenitor cells; OECs are differentiated cells, and so are unlikely to reside in the progenitor cell fraction. Finally, when OEC cultures established in our lab were grown under our culture conditions they did not survive well, and did not generate cells of other lineages. Thus our cultures are true progenitor fraction cultures, and the remyelination we found can be attributed to a subset of neural progenitor cells.

### Schwann cell-like remyelination

The adult derived OB progenitor fraction cells remyelinated rat spinal cord with a peripheral type of myelin that labels for P0. Generation of peripheral myelin on demyelinated CNS axons after transplantation of centrally derived progenitors has been described. Human neural progenitors, and embryonic stem cells have given this result [4], [45]. Committed oligodendrocyte progenitors isolated from optic nerve [46] and rat spinal cord [45] also generate peripheral myelin.

Our cells are derived from the adult rat progenitor fraction. Multipotent adult-derived rat progenitor cells isolated from the spinal cord have recently been isolated and grafted into the X-EB model [15], and the results of this work are similar to ours, with central progenitors generating a peripheral type of myelin, indicating that progenitors of very different origins may yield cells of the same phenotypes *in vivo*, perhaps because they respond in the same way to available environmental cues in the graft zone. The lesion itself may play a role in the pattern of myelination observed.

Our lesion type, and time course for analysis of grafted spinal cords was chosen to limit the possibility of endogenous remyelination in lesioned animals. Our previous electrophysiological results show that demyelination by EB alone without X-irradiation or cell grafting ultimately results in endogenous remyelination and a significant improvement in conduction velocity over the course of a few weeks in rat [44], whereas the addition of X-irradiation allows for a prolonged, persistent aglial zone [20]. OB cells were grafted three days post-EB lesion and the spinal cords were evaluated three weeks after grafting, so that there would be no possibility of migration of endogenous glia or glial progenitors to the demyelination site during the experiment.

The OB cells did not differentiate into neurons or astrocytes after transplantation into the X-EB lesion; the dominant differentiation was into a peripheral myelin-forming cell. The X-EB lesion is an aglial lesion where both astrocytes and oligodendrocytes are killed [47] (for a review see [48]). The loss of astrocytes seems to have a profound impact on the potential for grafted cells to develop into oligodendrocytes. When Talbott et al., [49] grafted oligodendrocyte precursors into the X-EB lesioned spinal cord, those cells differentiated into Schwann cell-like peripheral myelinating cells. However, when oligodendrocyte precursors were grafted along with astrocytes, a natural source of noggin that inhibits bone morphogenetic protein (BMP) signaling *in vivo*, they blocked the formation of P0^+^ myelin, and some grafted cells at the edge of the lesion differentiated into central myelinating MBP^+^ cells [45]. To confirm the role of noggin/BMP in differentiation of grafted cells, the group transduced oligodendrocyte progenitors *in vitro* to produce noggin, and grafted noggin^+^ cells into the X-EB lesion. Again, they blocked the P0^+^ myelin from forming [45]. They concluded that Schwann cell-like differentiation of oligodendrocyte precursors derived from adult spinal cord is BMP dependent. Interestingly, when they grafted “glial-restricted progenitors” into the lesion, these cells did not generate P0^+^ myelin, and instead differentiated into GFAP^+^ astrocytes [45].

Our data suggest that adult-derived cells from the OB progenitor fraction - a mixed progenitor population - default to primarily a peripheral type of myelinating cell when transplanted into an aglial zone in the spinal cord, as do cells of spinal cord and optic nerve origin when grafted into the same lesion [45], [46]. These results emphasize the importance of environmental cues in cell fate realization in our OB cells, similar to observations made of oligodendrocyte precursors [49], and progenitors isolated from different brain regions [2]. Moreover, the results indicate that OB cells from the progenitor fraction can be expanded in monolayer cultures and used for transplantation in demyelinating disorders.

## References

[1] Palmer TD, Markakis EA, Willhoite AR, Safar F, Gage FH (1999). Fibroblast growth factor-2 activates a latent neurogenic program in neural stem cells from diverse regions of the adult CNS.. J Neurosci.

[2] Markakis EA, Palmer TD, Randolph-Moore L, Rakic P, Gage FH (2004). Novel neuronal phenotypes from neural progenitor cells.. J Neurosci.

[3] Nait-Oumesmar B, Decker L, Lachapelle F, Avellana-Adalid V, Bachelin C (1999). Progenitor cells of the adult mouse subventricular zone proliferate, migrate and differentiate into oligodendrocytes after demyelination.. Eur J Neurosci.

[4] Akiyama Y, Honmou O, Kato T, Uede T, Hashi K (2001). Transplantation of clonal neural precursor cells derived from adult human brain establishes functional peripheral myelin in the rat spinal cord.. Exp Neurol.

[5] Vroemen M, Aigner L, Winkler J, Weidner N (2003). Adult neural progenitor cell grafts survive after acute spinal cord injury and integrate along axonal pathways.. Eur J Neurosci.

[6] Hardison JL, Nistor G, Gonzalez R, Keirstead HS, Lane TE (2006). Transplantation of glial-committed progenitor cells into a viral model of multiple sclerosis induces remyelination in the absence of an attenuated inflammatory response.. Exp Neurol.

[7] Reynolds BA, Tetzlaff W, Weiss S (1992). A multipotent EGF-responsive striatal embryonic progenitor cell produces neurons and astrocytes.. J Neurosci.

[8] Lois C, Alvarez-Buylla A (1993). Proliferating subventricular zone cells in the adult mammalian forebrain can differentiate into neurons and glia.. Proc Natl Acad Sci U S A.

[pone.0007260-Craig1] Craig CG, Tropepe V, Morshead CM, Reynolds BA, Weiss S (1996). In vivo growth factor expansion of endogenous subependymal neural precursor cell populations in the adult mouse brain.. J Neurosci.

[10] Alvarez-Buylla A, Herrera DG, Wichterle H (2000). The subventricular zone: source of neuronal precursors for brain repair.. Prog Brain Res.

[11] Liu Z, Martin LJ (2003). Olfactory bulb core is a rich source of neural progenitor and stem cells in adult rodent and human.. J Comp Neurol.

[12] Luskin MB (1993). Restricted proliferation and migration of postnatally generated neurons derived from the forebrain subventricular zone.. Neuron.

[13] Doetsch F, García-Verdugo JM, Alvarez-Buylla A (1997). Cellular composition and three-dimensional organization of the subventricular germinal zone in the adult mammalian brain.. J Neurosci.

[14] Gritti A, Bonfanti L, Doetsch F, Caille I, Alvarez-Buylla A (2002). Multipotent neural stem cells reside into the rostral extension and olfactory bulb of adult rodents.. J Neurosci.

[15] Mothe AJ, Tator CH (2008). Transplanted neural stem/progenitor cells generate myelinating oligodendrocytes and Schwann cells in spinal cord demyelination and dysmyelination.. Exp Neurol.

[16] Babu H, Cheung G, Kettenmann H, Palmer TD, Kempermann G (2007). Enriched monolayer precursor cell cultures from micro-dissected adult mouse dentate gyrus yield functional granule cell-like neurons.. PLoS One.

[17] Palmer TD, Takahashi J, Gage FH (1997). The adult rat hippocampus contains primordial neural stem cells.. Mol Cell Neurosci.

[18] Gage FH, Coates PW, Palmer TD, Kuhn HG, Fisher LJ (1995). Survival and differentiation of adult neuronal progenitor cells transplanted to the adult brain.. Proc Natl Acad Sci U S A.

[19] Sasaki M, Lankford KL, Zemedkun M, Kocsis JD (2004). Identified olfactory ensheathing cells transplanted into the transected dorsal funiculus bridge the lesion and form myelin.. J Neurosci.

[20] Blakemore WF, Patterson RC (1978). Suppression of remyelination in the CNS by X-irradiation.. Acta Neuropathol (Berl).

[21] Honmou O, Felts PA, Waxman SG, Kocsis JD (1996). Restoration of normal conduction properties in demyelinated spinal cord axons in the adult rat by transplantation of exogenous Schwann cells.. J Neurosci.

[22] Sasaki M, Black JA, Lankford KL, Tokuno HA, Waxman SG (2006). Molecular reconstruction of nodes of Ranvier after remyelination by transplanted olfactory ensheathing cells in the demyelinated spinal cord.. J Neurosci.

[23] Sasaki M, Hains BC, Lankford KL, Waxman SG, Kocsis JD (2006). Protection of corticospinal tract neurons after dorsal spinal cord transection and engraftment of olfactory ensheathing cells.. Glia.

[24] Palmer TD, Takahashi J, Gage FH (1997). The adult rat hippocampus contains primordial neural stem cells.. Mol Cell Neurosci.

[25] Greenfield S, Brostoff S, Eylar EH, Morell P (1973). Protein composition of myelin of the peripheral nervous system.. J Neurochem.

[26] Lankford KL, Sasaki M, Radtke C, Kocsis JD (2008). Olfactory ensheathing cells exhibit unique migratory, phagocytic, and myelinating properties in the X-irradiated spinal cord not shared by Schwann cells.. Glia.

[27] Pagano SF, Impagnatiello F, Girelli M, Cova L, Grioni E (2000). Isolation and characterization of neural stem cells from the adult human olfactory bulb.. Stem Cells.

[28] Bayer SA (1983). 3H-thymidine-radiographic studies of neurogenesis in the rat olfactory bulb.. Exp Brain Res.

[29] Westergaard E (1970). The lateral cerebral ventricles and the ventricular walls.. Thesis, University of Aarhus, Denmark.

[30] Kornack DR, Rakic P (2001). The generation, migration, and differentiation of olfactory neurons in the adult primate brain.. Proc Natl Acad Sci U S A.

[31] Markakis EA, Gage FH (1999). Adult-generated neurons in the dentate gyrus send axonal projections to field CA3 and are surrounded by synaptic vesicles.. J Comp Neurol.

[32] Alvarez-Buylla A, Garcia-Verdugo JM (2002). Neurogenesis in adult subventricular zone.. J Neurosci.

[33] Kondo T, Raff M (2000). Oligodendrocyte precursor cells reprogrammed to become multipotential CNS stem cells.. Science.

[34] Hack MA, Saghatelyan A, de Chevigny A, Pfeifer A, Ashery-Padan R (2005). Neuronal fate determinants of adult olfactory bulb neurogenesis.. Nat Neurosci.

[35] Petreanu L, Alvarez-Buylla A (2002). Maturation and death of adult-born olfactory bulb granule neurons: role of olfaction.. J Neurosci.

[36] Franklin RJ, Gilson JM, Franceschini IA, Barnett SC (1996). Schwann cell-like myelination following transplantation of an olfactory bulb-ensheathing cell line into areas of demyelination in the adult CNS.. Glia.

[37] Imaizumi T, Lankford KL, Waxman SG, Greer CA, Kocsis JD (1998). Transplanted olfactory ensheathing cells remyelinate and enhance axonal conduction in the demyelinated dorsal columns of the rat spinal cord.. J Neurosci.

[38] Barnett SC, Alexander CL, Iwashita Y, Gilson JM, Crowther J (2000). Identification of a human olfactory ensheathing cell that can effect transplant-mediated remyelination of demyelinated CNS axons.. Brain.

[39] Sasaki M, Li B, Lankford KL, Radtke C, Kocsis JD (2007). Remyelination of the injured spinal cord.. Prog Brain Res.

[40] Doucette JR (1984). The glial cells in the nerve fiber layer of the rat olfactory bulb.. Anat Rec.

[41] Chuah MI, Au C (1993). Cultures of ensheathing cells from neonatal rat olfactory bulbs.. Brain Res.

[42] Ramon-Cueto A, Nieto-Sampedro M (1992). Glial cells from adult rat olfactory bulb: immunocytochemical properties of pure cultures of ensheathing cells.. Neuroscience.

[43] Au W, Treloar HB, Greer CA (2002). Sublaminar organization of the mouse olfactory bulb nerve layer.. J Comp Neurol.

[44] Akiyama Y, Lankford K, Radtke C, Greer CA, Kocsis JD (2004). Remyelination of spinal cord axons by olfactory ensheathing cells and Schwann cells derived from a transgenic rat expressing alkaline phosphatase marker gene.. Neuron Glia Biol.

[45] Keirstead HS, Nistor G, Bernal G, Totoiu M, Cloutier F (2005). Human embryonic stem cell-derived oligodendrocyte progenitor cell transplants remyelinate and restore locomotion after spinal cord injury.. J Neurosci.

[46] Groves AK, Barnett SC, Franklin RJM, Crang AJ, Mayer M (1993). Repair of demyelinated lesions by transplantation of purified O-2A progenitor cells.. Nature.

[47] Graça DL, Blakemore WF (1986). Delayed remyelination in rat spinal cord following ethidium bromide injection.. Neuropathol Appl Neurobiol Nov-Dec;.

[48] Blakemore WF, Franklin RJ (2008). Remyelination in experimental models of toxin-induced demyelination.. Curr Top Microbiol Immunol.

[49] Talbott JF, Cao Q, Enzmann GU, Benton RL, Achim V (2006). Schwann cell-like differentiation by adult oligodendrocyte precursor cells following engraftment into the demyelinated spinal cord is BMP-dependent.. Glia.

